# Inhibition of glycogen synthase kinase-3 enhances NRF2 protein stability, nuclear localisation and target gene transcription in pancreatic beta cells

**DOI:** 10.1016/j.redox.2024.103117

**Published:** 2024-03-07

**Authors:** Chinmai Patibandla, Lidy van Aalten, Albena T. Dinkova-Kostova, Tadashi Honda, Antonio Cuadrado, Raquel Fernández-Ginés, Alison D. McNeilly, John D. Hayes, James Cantley, Calum Sutherland

**Affiliations:** a Division of Cellular & Systems Medicine, James Arnott Drive, Ninewells Hospital and Medical School, University of Dundee, Dundee, DD1 9SY, Scotland, United Kingdom; b Institute of Chemical Biology and Drug Discovery, Stony Brook University, Stony Brook, NY, USA; c Department of Chemistry, Stony Brook University, Stony Brook, NY, USA; d Instituto de Investigaciones Biomédicas Sols-Morreale UAM-CSIC, Instituto de Investigación Sanitaria La Paz (IdiPaz) and Department of Biochemistry, Faculty of Medicine, Autonomous University of Madrid, Madrid, Spain; e Centro de Investigación Biomédica en Red Sobre Enfermedades Neurodegenerativas (CIBERNED), ISCIII, Madrid, Spain

**Keywords:** NRF2, GSK3, β-TrCP, β cells, Islets

## Abstract

Accumulation of reactive oxygen species (i.e., oxidative stress) is a leading cause of beta cell dysfunction and apoptosis in diabetes. NRF2 (NF-E2 p45-related factor-2) regulates the adaptation to oxidative stress, and its activity is negatively regulated by the redox-sensitive CUL3 (cullin-3) ubiquitin ligase substrate adaptor KEAP1 (Kelch-like ECH-associated protein-1). Additionally, NRF2 is repressed by the insulin-regulated Glycogen Synthase Kinase-3 (GSK3). We have demonstrated that phosphorylation of NRF2 by GSK3 enhances β-TrCP (beta-transducin repeat-containing protein) binding and ubiquitylation by CUL1 (cullin-1), resulting in increased proteasomal degradation of NRF2. Thus, we hypothesise that inhibition of GSK3 activity or β-TrCP binding upregulates NRF2 and so protects beta cells against oxidative stress. We have found that treating the pancreatic beta cell line INS-1 832/13 with the KEAP1 inhibitor TBE31 significantly enhanced NRF2 protein levels. The presence of the GSK3 inhibitor CT99021 or the β-TrCP-NRF2 protein-protein interaction inhibitor PHAR, along with TBE31, resulted in prolonged NRF2 stability and enhanced nuclear localisation (P < 0.05). TBE31-mediated induction of NRF2-target genes encoding NAD(P)H quinone oxidoreductase 1 (*Nqo1*), glutamate-cysteine ligase modifier (*Gclm*) subunit and heme oxygenase (*Hmox1*) was significantly enhanced by the presence of CT99021 or PHAR (P < 0.05) in both INS-1 832/13 and in isolated mouse islets. Identical results were obtained using structurally distinct GSK3 inhibitors and inhibition of KEAP1 with sulforaphane. In summary, we demonstrate that GSK3 and β-TrCP/CUL1 regulate the proteasomal degradation of NRF2, enhancing the impact of KEAP1 regulation, and so contributes to the redox status of pancreatic beta cells. Inhibition of GSK3, or β-TrCP/CUL1 binding to NRF2 may represent a strategy to protect beta cells from oxidative stress.

## Highlights

• We provide the first evidence that inhibition of GSK3 activity enhances NRF2 function in beta cells and isolated islets.
• Transient knock down of GSK3 but not overexpression affects NRF2 protein levels.
• Inhibition of βTrCP-NRF2 protein-protein interaction has similar effects on NRF2 function as GSK3 inhibition.

## 1 Introduction

Diabetes Mellitus, a complex metabolic disorder affecting hundreds of millions worldwide, poses a major public health challenge [1]. Central to the pathogenesis of this condition is the pancreatic beta cell, which is required for the endocrine response to rising blood glucose levels, delivering appropriate insulin secretion to maintain glucose homeostasis. However, beta cell function can be disrupted by excessive accumulation of reactive oxygen species (ROS) [2], which are formed as by-products of metabolism. During the pathogenesis of type 2 diabetes, beta cells often hyper-secrete insulin to compensate for declining tissue sensitivity to insulin and this is associated with increased production of ROS [3]. Over time, an imbalance between ROS production and the capacity of the endogenous antioxidant defence system to ablate ROS can lead to oxidative stress, endoplasmic reticulum stress and insulin protein misfolding, with resultant loss of insulin secretion and eventually loss of beta cell mass [4]. This fact explains the need for insulin treatment and the associated risks that accompany medication. It is, therefore, clinically important to combat any decline in beta cell function.

NRF2 (NF-E2 p45-related factor 2) is a key transcription factor that serves as a molecular sentinel against oxidative stress and inflammation [5,6]. KEAP1 (Kelch-like ECH-associated protein 1) is a cytoplasmic protein that acts as a negative regulator of NRF2 by serving as a substrate adaptor for the CUL3 (cullin-3) E3 ubiquitin ligase complex. It functions as a sensor of redox perturbation [7]. Under normal conditions, KEAP1 binds NRF2 and facilitates its degradation through the ubiquitin-proteasome system [8,9]. When cells experience oxidative stress, ROS and secondary reactive aldehydes created by ROS, modify certain cysteines within KEAP1, preventing it from targeting NRF2 for proteasomal degradation. Consequently, *de novo* synthesised NRF2 bypasses KEAP1 and accumulates in the nucleus where it heterodimerises with a small MAF (musculoaponeurotic fibrosarcoma) protein before binding to antioxidant response element (ARE) sequences in gene promoters, whereupon it induces cytoprotective genes encoding antioxidant enzymes, detoxification enzymes, and anti-inflammatory molecules, that work together to neutralize ROS and restore cellular redox balance [6]. In beta cells, NRF2 activation protects them from oxidative damage and preserves their insulin-secreting capacity [10]. Enhanced NRF2 activity in beta cells promotes cell survival and improves insulin secretion under conditions of oxidative stress [11,12]. On the other hand, impaired NRF2 signalling is associated with beta cell dysfunction and increased susceptibility to ROS-induced damage, contributing to the development of diabetes mellitus [12].

Previously, we have shown that GSK3 activity regulates NRF2 by phosphorylating the DSGIS motif located within the Neh6 (NRF2-ECH homology 6) domain of NRF2, thus promoting proteasomal degradation directed by β-TrCP (beta-transducin repeat-containing protein)-CUL1, a mechanism that has been reported in human and mouse tumour cells [13,14]. In vivo experiments with a mouse strain in which the two serines in the DSGIS motif in Neh6 were mutated to non-phosphorylatable alanines have shown enhanced NRF2 function in animals with suppressed KEAP1 activity [15]. This transgenic model confirms that phosphorylation of DSGIS motif cooperates with KEAP1 to suppress NRF2 activity under physiological conditions [15].

GSK3 is a serine/threonine protein kinase that plays a critical role in various cellular processes, such as glycogen metabolism, cell signalling, gene transcription, cell division, and cell survival [16]. GSK3 exists in two highly similar isoforms, GSK-3α and GSK-3β, which share about 90% sequence similarity. They are encoded by separate genes and have different tissue distributions [17]. Both isoforms of GSK3 are expressed in beta cells and are downstream targets of the insulin receptor signalling pathway. Insulin induces an inhibitory phosphorylation (at Ser21/Ser9 in GSK3α/β [18,19]) through activation of phosphatidylinositol 3-kinase and its downstream target protein kinase B (PKB)/Akt [20]. Other signalling pathways known to regulate PKB/Akt (eg growth factors) and other protein kinases (eg p90RSK, p70S6K, CAMK-II and PKA [18,19,21,22]) are reported to inhibit GSK3 through phosphorylation of these same serine residues, in specific physiological circumstances. Other phosphorylation sites can also modulate GSK3 activity. For example, phosphorylation of Tyr279/216 in GSK3α/β is required for inherent activity and is thought to be stoichiometrically auto-phosphorylated by GSK3 in most cells [23]. However, there is some evidence that it can be dynamically regulated to modify GSK3 activity, at least in neurons [24]. The other key signalling pathway that utilises GSK3 is the canonical Wnt pathway [25]. Wnts regulate GSK3 by disrupting its association with the axin/β-catenin complex, thereby reducing phosphorylation of other complex components and stabilising β-catenin. These two pools of GSK3 appear to be distinctly regulated in cells [26]. In type 2 diabetes, enhanced GSK3 activity is associated with the development of insulin resistance [27]. In beta cells, GSK3 regulates several transcription factors, insulin secretion and proliferation [[28], [29], [30]]. GSK3 levels are highly upregulated in beta cells isolated from patients with type 2 diabetes [31]. Thus, we hypothesised that pathologically high GSK3 activity would impair NRF2-target gene expression in beta cells and contribute to loss of redox homeostasis during the pathogenesis of diabetes by increasing repression of NRF2 via the β-TrCP/GSK3 axis. Targeting the β-TrCP/GSK3 axis would upregulate NRF2, improve beta cell function, slow the progression of type 2 diabetes and protect against the requirement for insulin treatment.

## 2 Materials and methods

### 2.1 Chemicals

Sulforaphane (SFN) was purchased from Cambridge Bioscience and LY2090314 from Stratech. The synthesis of CT99021 and TBE31 has been previously described [32,33], and the synthesis of PHAR and its negative control are described elsewhere [34]. All stocks were prepared by dissolving in DMSO.

### 2.2 Antibodies

Details of all primary and secondary antibodies are provided in Table 1, Table 2.

**Table 1:** List of primary antibodies used in the study.

Antibody	Manufacturer	Catalogue number	Lot Number	Host	Dilution
NRF2	Cell Signalling Technologies	20733	Lot 1	Rabbit	1:1000
NQO1	Abcam	Ab80588	GR3317475-20	Rabbit	1:5000
KEAP1	Millipore	MABS514	3474155	Rat	1:1000
Phospho GSK3	Cell Signalling Technologies	8566	Lot 3	Rabbit	1:1000
Total GSK3	Cell Signalling Technologies	5676	Lot 7	Rabbit	1:1000
Phospho CRMP2	Inhouse			Sheep	1:1000
Total CRMP2	Cell Signalling Technologies	9393	Lot 1	Rabbit	1:1000
β-Tubulin	Cell Signalling Technologies	2128	Lot 11	Rabbit	1:1000
Lamin A/C	Cell Signalling Technologies	2032	Lot 6	Rabbit	1:1000
TBP	Abcam	Ab300656	GR3459461-3	Mouse	1:1000
HSP90α	Cell Signalling Technologies	8165	Lot 1	Rabbit	1:1000
GAPDH	Cell Signalling Technologies	5174	Lot 8	Rabbit	1:1000

**Table 2:** List of Secondary antibodies used in the study.

Secondary Antibody	Manufacturer	Catalogue number	Lot Number	Host	Dilution
Anti-Rat IRDye® 800CW	LI-COR	926-32219	D01118-15	Goat	1:5000
Anti-Rabbit IRDye® 800CW	LI-COR	926-32211	D30110-05	Goat	1:5000
Anti-Mouse Alexa Fluor™ 680	Invitrogen	A21057	2400916	Goat	1:5000
Anti-Rabbit HRP	Invitrogen	31460	XB339163	Goat	1:5000
Anti-Sheep HRP	Invitrogen	31480	XF3600072A	Rabbit	1:5000

### 2.3 Cell culture

INS-1 832/13 cells are a rat immortalised beta cell line that exhibits glucose-stimulated insulin secretion [35,36]. They were maintained in growth media containing RPMI-1640 medium supplemented with 5% FBS, 1% Pen/Strep, 50 μM β-mercaptoethanol, 1 mM sodium pyruvate, 10 mM HEPES, and 2 mM L-glutamine in a humidified atmosphere of 5% CO_2_/95% air at 37 °C. Cells were seeded in 6-well plates (2 × 10^6^ cells per well) and cultured for 48 h in growth media prior to incubation with compounds (as detailed in figure legends) in 0.5% FBS containing media for a further 24 h. For isolation of cytoplasmic and nuclear extracts cells were seeded in T75 flasks (4 × 10^6^ cells per flask) and cultured for 48 h in full growth media prior to 24 h treatment with compounds (as detailed in figure legends) in 0.5% FBS containing media.

### 2.4 CRISPR KO of Keap1

Lentiviral CRISPR all-in-one vectors expressing either non-targeting control/Scramble gRNA (GCTTAGTTACGCGTGGACGA) [37] or rat Keap1 gRNA (GGCCGCAACAACTCGCCGGA) along with Cas9, both from the U6 promoter, with a puromycin selection gene expressed from an EFS promoter, were designed and purchased from VectorBuilder Inc., USA. The gRNA targets the coding region of Keap1 on Chromosome 8, NC_051343.1, 19772258-19772277 which corresponds to amino acids 375-381 in the Kelch domain (NRF2 binding domain) of the protein. The INS-1 832/13 cells were seeded in a 6-well plate (1 × 10^6^ cells/well) and cultured for 48 h prior to lentiviral infection with 5 μl of either control or Keap1 gRNA (both stocks at 4.5*10^8^TU/ml). Media was changed at 24 h and puromycin selection media (full growth media without Pen/Strep and with 3 μg/ml puromycin) was added 72 h post-infection. Cells were cultured in selection media every second passage to maintain selection.

### 2.5 GSK3 knock-down and overexpression

ON-TARGETplus (SMARTpool) Small interfering RNA targeting rat GSK3α, GSK3β or non-targeting controls and transfection reagent DharmaFECT 1 were purchased from Horizondiscovery, UK. Cells were seeded at a density of 5 × 10^5^ cells/well in a 6-well plate and cultured for 48 h. For transient transfection, the media was changed to Pen/Strep-free complete media and transfected with 5 nmol/l of either control, GSK3α or GSK3β siRNA using DharmaFECT 1 transfection reagent according to the manufacturer's protocol. Media was changed to complete media 24 h post-transfection and RNA or protein was extracted 72 h post-transfection.

For overexpression of GSK3, cells were seeded and cultured in 6-well plates for 48 h and infected with adenovirus expressing GFP, GSK3α or GSK3β ORF under the CMV promoter. The culture media was changed to Complete media after 2 h and cultured for a further 24 h prior to extraction of RNA or protein. Adenoviruses expressing GFP or GSK3α (10^10^ PFU/ml) were purchased from VectorBuilder, USA. The adenovirus expressing GSK3β has been described previously [32]. Protein levels of GSK3α or GSK3β were measured by immunoblot at 6 h, 12 h and 24 h post-infection (Supplementary Fig 1). The optimal increase in both GSK3α and GSK3β protein levels was after 24 h, so this was used for all subsequent experiments.

### 2.6 Cellular protein extraction

Protein was extracted from cells in SDS lysis buffer [50 mM Tris HCl pH 6.8, 2% (w/v) SDS and 10% Glycerol (v/v)], prior to sonication for 30 s (5-sec pulses) at 20% amplitude, and the protein content quantified using Pierce™ BCA Protein Assay Kit (ThermoFisher Scientific, UK). Cytoplasmic and nuclear fractions were extracted using NE-PER™ Extraction Reagents (ThermoFisher Scientific, UK) as per the manufacturer's instructions. The quality of fraction preparations was checked by immunoblotting with cytoplasmic (HSP90α, GAPDH) or nuclear (Lamin A/C) markers (Supplementary Fig. 2).

### 2.7 Western blotting

All protein lysates were prepared in SDS loading buffer [50 mM Tris HCl pH 6.8, 2% (w/v) SDS and 10% glycerol (v/v), 1% β-mercaptoethanol, 0.02% bromophenol blue] and heated to 70 °C for 5 min before loading. Whole cell (20 μg protein), cytoplasmic (20 μg protein) or nuclear (10 μg protein) lysates were separated by SDS-PAGE on 4% stacking-12% resolving polyacrylamide gels (Mini-PROTEAN Precast Gel system, BioRad, UK). Proteins were transferred to 0.45 μm nitrocellulose (Amersham™ Protran®, Cytiva, Germany) and membranes were blocked with 5% fatty acid-free bovine serum albumin prior to overnight incubation with primary antibodies in blocking buffer (details in Table 1) at 4 °C as previously described [38]. NRF2 and pCRMP2 were detected by the ECL method (detailed in Table 2) and all other primary antibodies were detected and quantified using Alexa Fluor™ 680 or IRDye® 800CW-tagged secondary antibodies on a Licor Odyssey CLx (Analysed by Image Studio Lite Version 5.2).

### 2.8 qPCR

RNA was extracted from INS-1 832/13 cells after treatment using High Pure RNA Tissue Kit (Roche, UK). cDNA was generated from 500 ng of RNA using a High-Capacity cDNA Reverse Transcription Kit (ThermoFisher Scientific, UK) as per the manufacturer's instructions. cDNA was diluted in water 1:10 prior to real-time PCR analysis on an Applied Biosystems Quantstudio 7 Flex real-time PCR instrument using SolisFAST® Probe qPCR Mix (Solis Biodyne, Estonia). Predesigned rat and mouse TaqMan™ Assays (ThermoFisher Scientific, UK) used in the study are listed in Table 3.

**Table 3:** List of probe based qPCR assays used in the study.

gene	Accession Number	Catalogue number	Amplicon Size (bp)	Assay Location
Rat *Nrf2/Nfe2l2*	NM_031789.2	Rn00582415_m1	104	379
Rat *Nqo1*	NM_017000.3	Rn00566528_m1	90	607
Rat *Gclc*	NM_012815.2	Rn00689048_m1	65	505
Rat *Gclm*	NM_017305.2	Rn00568900_m1	80	251
Rat *Hmox1*	NM_012580.2	Rn00561387_m1	132	263
Rat *Txnrd1*	NM_031614.2	Rn01503798_m1	94	1528
Rat *Hprt1*	NM_012583.2	Rn01527840_m1	64	673
Mouse *Nrf2/Nfe2l2*	NM_010902.3	Mm00477784_m1	61	279
Mouse *Nqo1*	NM_008706.5	Mm01253561_m1	81	545
Mouse *Gclc*	NM_010295.2	Mm00802658_m1	78	1974
Mouse *Gclm*	NM_008129.4	Mm01324400_m1	87	963
Mouse *Hmox1*	NM_010442.2	Mm00516005_m1	69	279
Mouse *Txnrd1*	NM_015762.2	Mm00443675_m1	63	1558
Mouse *Hprt*	NM_013556.2	Mm03024075_m1	131	276

### 2.9 Mouse pancreatic islet isolation and inhibitor exposure

Islets were isolated as described previously [39] from mice maintained in conditions fully compliant with the ARRIVE guidelines and all experiments were carried out in accordance with the U.K. Animals (Scientific Procedures) Act, 1986 and associated guidelines, EU Directive 2010/63/EU for animal experiments. Briefly, 12-week-old male C57BL/6J mice (pancreata were perfused with collagenase and thermolysin (Liberase T-flex, Roche). After incubation at 37 °C for 16 min, tissue was mechanically disrupted, and islets were separated using Ficoll-Paque plus gradient (GE Healthcare, UK). Isolated islets were cultured overnight in full growth media (RPMI-1640 medium supplemented with 10% FBS, 1% Pen/Strep, 15 mM HEPES, and 2 mM L-glutamine) prior to incubation with inhibitors or carrier (DMSO) for 24 h in the same media before extracting RNA.

### 2.10 Statistical analysis

Results are presented as mean ± S.E.M. Data were analysed using GraphPad PRISM® software (version 10.0.1) with one-way ANOVA used to compare more than 2 groups (Sidak's or Dunnett's post hoc test) with a significance threshold of p < 0.05.

## 3 Results

### 3.1 GSK3 inhibition increases the abundance of NRF2 protein in the nucleus of pancreatic beta cells

**Fig. 1:**
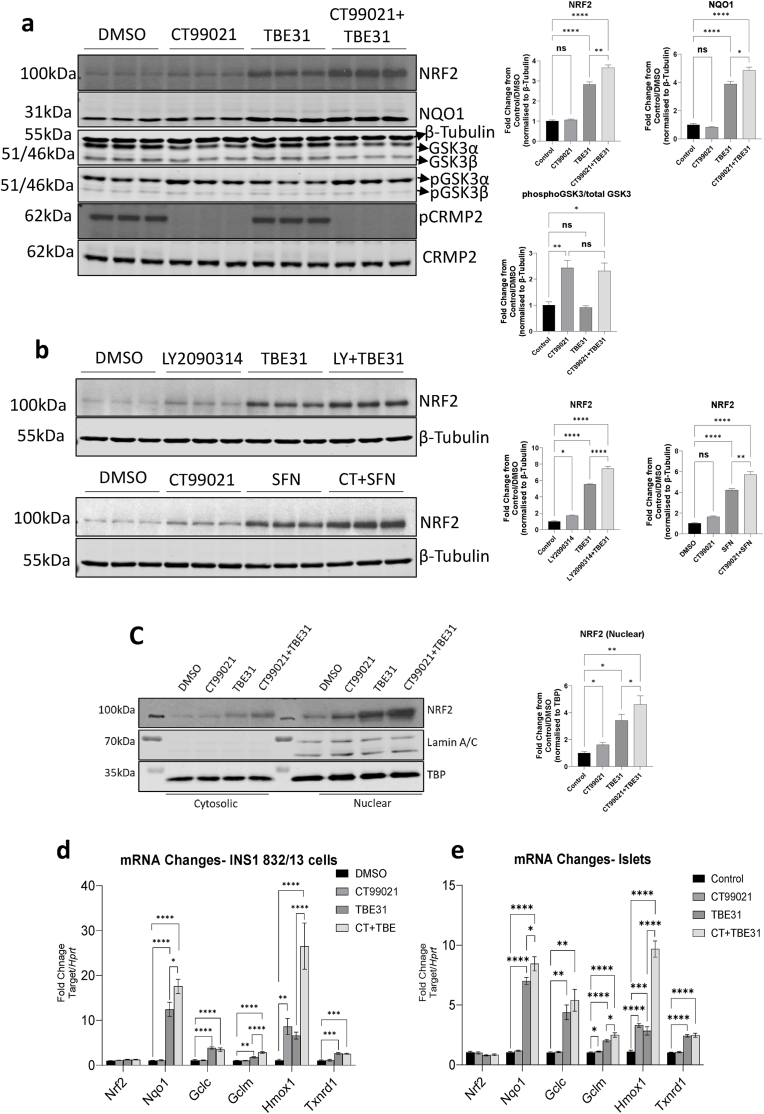
**a, b)** INS-1 832/13 cells were treated for 24 h with DMSO, the GSK3 inhibitors CT99021 (5 μmol/l) or LY2090314 (20 nmol/l), the KEAP1 inhibitors TBE31 (50 nmol/l) or SFN (5 μmol/l), or combinations (CT99021+TBE31, LY2090314+TBE31 or CT99021+SFN). Whole-cell protein lysates from three independent experiments (20 μg protein/lane) were analysed by western blotting using β-tubulin as a loading control. **c)** INS-1 832/13 cells were treated for 24 h with DMSO, CT99021 (5 μmol/l), TBE31 (50 nmol/l) or CT99021 +TBE31, and then cytoplasmic and nuclear protein extracts were prepared. Samples of either cytoplasmic fractions (20 μg protein/lane) or nuclear fractions (10 μg protein/lane) were separated by SDS-PAGE and NRF2 protein levels analysed with TBP as loading control. Data from seven independent experiments were analysed by paired ANOVA with Sidak's post hoc test. **d,e)** RNA from INS-1 832/13 cells (n = 3) or islets isolated from 12-week-old male C57BL/6J mice (n = 6) were extracted after 24 h treatment with the agents described above and expression of NRF2-target genes analysed by qPCR. Data are presented as mean and standard error and analysed by one-way ANOVA with Sidak's post hoc test. *P < 0.05, **P < 0.01, ***P < 0.001, ****P < 0.0001.

To investigate the importance of GSK3 activity on steady-state levels of NRF2 protein, INS-1 832/13 cells were treated with the GSK3 inhibitor CT99021 (5 μmol/l) [40], with or without the KEAP1 inhibitor TBE31 (50 nmol/l) for 24 h (Fig. 1); inhibition of GSK3 was confirmed by significant loss of phosphorylation of the validated GSK3 target CRMP2 [41] at Ser509 (Fig. 1a). Incubating cells with CT99021 alone had no significant effect on the basal levels of NRF2 protein in total lysates from INS-1 832/13 cells (Fig. 1a). As expected, treatment with TBE31 for 24 h significantly enhanced steady-state levels of NRF2 protein in whole lysates (P < 0.0001). However, co-treatment with CT99021 and TBE31 enhanced NRF2 protein levels above that seen with TBE31 alone (P < 0.0001), indicating a co-operative effect between KEAP1 and GSK3 inhibition. This increase in NRF2 protein was maintained at 36 h of CT99021 treatment (Supplementary Fig. 3). Consistent with an increase in NRF2 activity, the protein product of the NRF2-regulated gene *Nqo1* was increased by TBE31 treatment and further augmented with CT99021 in combination with TBE31 (P < 0.0001). Meanwhile, CT99021 treatment alone did not alter NQO1 protein levels (Fig. 1a). Almost identical results were obtained when the structurally unrelated GSK3 inhibitor LY2090314 [42] and the alternative KEAP1 inhibitor SFN were used (Fig. 1b). In this case a small but significant increase in NRF2 abundance when INS-1 832/13 cells were treated with LY2090314 alone. Hence, treatment with LY2090314 or CT99021 in combination with TBE31 or SFN had co-operative effects on the abundance of NRF2 protein (P < 0.0001), consistent with an additional, KEAP1-independent suppression of NRF2 protein levels by GSK3.

Accumulation of NRF2 in the nucleus is required for the ARE-driven antioxidant response. Thus, we investigated whether inhibition of GSK3 increased NRF2 nuclear localisation (Fig. 1c). Treatment with CT99021 alone produced a small but significant increase in the nuclear localisation of NRF2 (P < 0.05). Consistent with whole-cell protein levels, nuclear NRF2 protein levels were significantly elevated by TBE31 alone, while incubation with TBE31 and CT99021 together further enhanced NRF2 protein levels (P < 0.05). We then measured mRNA levels of five known NRF2-target genes in both INS-1 832/13 cells and isolated mouse islets treated with these compounds (Fig. 1d & e). Treatment for 24 h with CT99021 alone had no significant effect on any of these NRF2 target genes in INS-1 832/13 cells or isolated islets (Fig. 1d). TBE31 treatment alone significantly enhanced the mRNA production for all NRF2-target genes examined, both in INS-1 832/13 cells and isolated islets, albeit with varying potency (except the *Hmox1* gene which exhibited high variance but a trend of induction). Consistent with the western blotting data above, CT99021 further enhanced the expression of *Nqo1* (P < 0.05), *Gclm* (P < 0.0001) and *Hmox1* (P < 0.01) over TBE31 treatment alone in INS-1 832/13 cells. In the mouse islets, CT99021 in combination with TBE31 enhanced *Nqo1* (P < 0.05) and *Hmox1* (P < 0.0001) expression when compared with TBE31 treatment alone. Interestingly, GSK3 inhibition had no impact on the TBE31 induction of *Txrnd1* or *Gclc* expression in INS-1 832/13 or in isolated islets.

### 3.2 The impact of transient knock-down of GSK3 isoforms on NRF2 activity

**Fig. 2:**
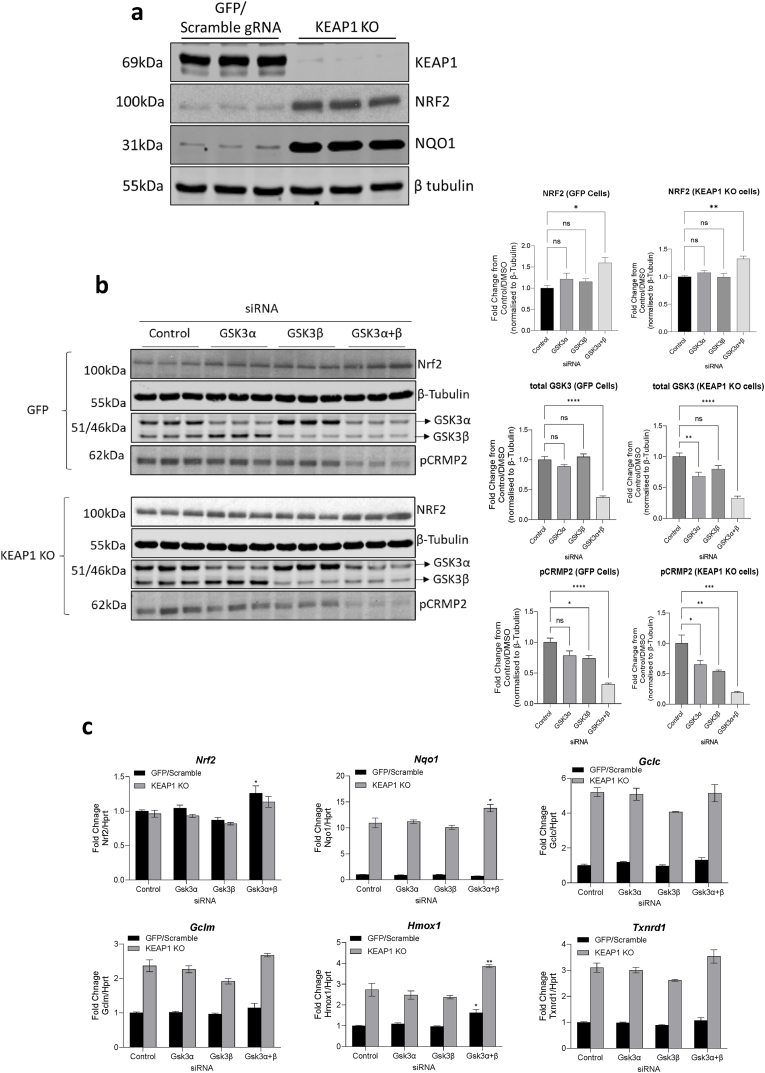
**a)** INS-1 832/13 stable cell lines lacking KEAP1 were generated by transfection with a lentiviral all-in-one plasmid containing either control/Scramble gRNA or rat KEAP1 gRNA along with Cas9, and a puromycin selection gene. Whole-cell lysates were extracted and KEAP1 deletion as well as subsequent enhanced NRF2 expression and activity (NQO1) were confirmed by western blotting. Tubulin was used as a loading control. **b)** GFP or KEAP1 KO cells were transfected with siRNA targeting GSK3α, GSK3β or both GSK3α+β. Seventy-two hr after transfection, whole cell lysates were extracted and analysed by western blot as shown. This confirmed GSK3α+β knockdown and the subsequent impact on phosphorylation of the GSK3 substrate CRMP2, along with NRF2 protein levels, assessed. Tubulin was used as loading control. The GSK3, pCRMP2 and NRF2 blots were quantified from 3 separate experiments run in duplicate and are presented relative to control as mean ± standard error, analysed by one-way ANOVA with Dunnett's post hoc test. **c)** KEAP1 KO or GFP expressing control cells were transfected with siRNA targeting GSK3α, GSK3β or both GSK3α+β. RNA was extracted 72h after transfection and NRF2-target gene expression was quantified by qPCR. Data are presented as mean ± standard error and analysed by one-way ANOVA with Dunnett's post hoc test. *P < 0.05, **P < 0.01, ***P < 0.001, ****P < 0.0001.

As an alternative to pharmacological manipulation, and since there are no isoform-specific GSK3 inhibitors available, we next examined if genetic reduction of one or both isoforms of GSK3 alters NRF2 protein levels in INS-1 832/13 cells in the presence or absence of KEAP1 regulation (Fig. 2). We first generated a KEAP1 knock-out INS-1 832/13 cell line using the CRISPR-Cas9 system, and confirmed deletion of KEAP1 by immunoblotting for KEAP1, NRF2 and NQO1 protein (Fig. 2a). GSK3α and GSK3β specific siRNA was used to transiently knock-down each target isoform individually, or both isoforms together, for comparison with cells exposed to non-targeting siRNA (control). After 72 h of siRNA transfection, GSK3 knock-down was confirmed by measuring GSK3 total protein levels and the phosphorylation of the GSK3 substrate CRMP2 (Fig. 2b). Reducing either isoform of GSK3 by 40–70% had no significant effect on NRF2 protein levels either in control or KEAP1 KO cells, but in each case was sufficient to reduce CRMP2 phosphorylation (Fig. 2b). Reduction of both isoforms together had a greater impact on pCRMP2 than the single knockdown (Fig. 2b). Interestingly, an upregulation of the alternate GSK3 isoform was observed when a single isoform was reduced, which implies compensation that makes it difficult to reduce total GSK3 activity through reduction in one isoform in these cells (Fig. 2b). NRF2 protein levels increased significantly when both GSK3 isoforms were reduced, implying total GSK3 activity was the key factor rather than isoform selective regulation of NRF2. Interestingly, this effect on NRF2 protein was seen in both control (P < 0.05) and KEAP1 KO cell lines (P < 0.01), demonstrating that GSK3 inhibits NRF2 independently of KEAP1 (Fig. 2b), though the effect is more evident in the KEAP1 deficient background as basal NRF2 is >10-times higher than in control cells (Fig. 2a). Consistent with the increase in NRF2 protein, the impact of GSK3 knockdown on induction of NRF2-target genes was only apparent when both GSK3 isoforms were reduced (Fig. 2c). Consistent with the experiments using pharmacological GSK3 inhibition, the enhancement of TBE31-induced NRF2-regulated mRNAs following genetic deletion of GSK3 was only seen for expression of *Nqo1* and *Hmox1* and only in KEAP1 KO cells (P < 0.05). Collectively, these results suggest the physiological consequences of GSK3 inhibition are more apparent when combined with KEAP1 inhibition (Fig. 2c).

### 3.3 The impact of transient overexpression of GSK3 isoforms on NRF2 activity

**Fig. 3:**
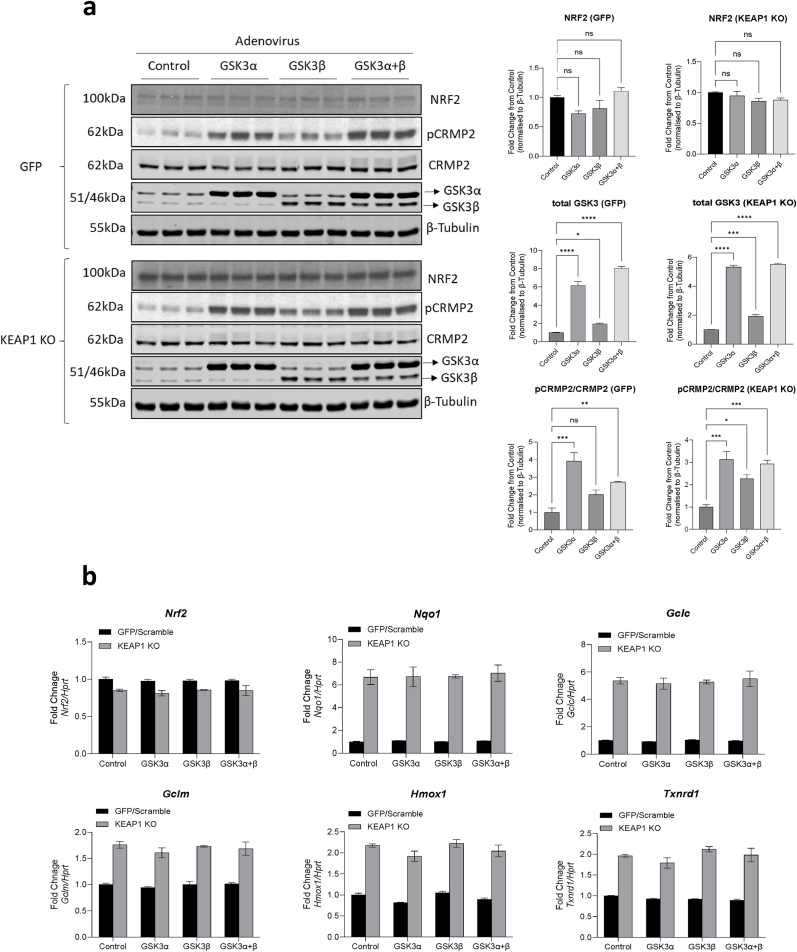
**a)** KEAP1 KO or GFP expressing control cells were transduced with adenoviral particles expressing GSK3α or GSK3β under the CMV promoter. Twenty-four hr later whole cell lysates were analysed for protein levels. This confirmed GSK3α+β overexpression and the subsequent impact on phosphorylation of the GSK3 substrate CRMP2, along with NRF2 protein levels, assessed. Tubulin was used as loading control. The GSK3, pCRMP2 and NRF2 blots were quantified from 3 separate experiments run in duplicate and are presented relative to control as mean ± standard error, analysed by one-way ANOVA with Dunnett's post hoc test. **b)** KEAP1 KO or GFP expressing control cells were transduced with GSK3α, GSK3β or GSK3α+β adenovirus, cultured for a further 24 h prior to RNA extraction and quantification of NRF2-target gene expression by qPCR. Data are presented as mean and standard error and analysed by one-way ANOVA with Dunnett's post hoc test. *P < 0.05, **P < 0.01, ***P < 0.001, ****P < 0.0001.

As GSK3 inhibition and genetic knock-down enhanced NRF2 function, we next examined if NRF2 protein levels and NRF2 activity could be antagonised by GSK3 overexpression (as seen in type 2 diabetes). We overexpressed the GSK3α or GSK3β isoforms alone, or together, in control or KEAP1 KO INS-1 832/13 cells (Fig. 3), and 24 h after adenoviral transduction, measured NRF2 protein. We confirmed that transduction of the adenoviral constructs into INS-1 832/13 cells enhanced GSK3 protein and that this increased the phosphorylation of the GSK3 substrate CRMP2 after 24 h (Fig. 3a). After 24 h infection with equivalent viral titres of each GSK3 adenovirus the levels of GSK3α were increased 5-6-fold and the levels of GSK3β doubled (n.b. the recombinant GSK3β has a Myc tag which means it has a slightly higher molecular mass than endogenous GSK3β). The phosphorylation of CRMP2 was enhanced in line with the degree of overexpression of GSK3α or GSK3β alone, or in combination (Fig. 3a). However, overexpression of GSK3α or GSK3β alone or in combination had no significant impact on NRF2 protein levels (Fig. 3a). Similarly, overexpression of one or both forms of GSK3 had no significant effect on expression of any of the NRF2-regulated genes either in control or KEAP1 KO INS-1 832/13 cells (Fig. 3b). These data suggest that there is little impact of this level of enhanced GSK3 activity on NRF2 function, at least acutely.

### 3.4 Specific inhibition of the NRF2-βTrCP interaction mirrors the outcomes of GSK3 deficiency

**Fig. 4:**
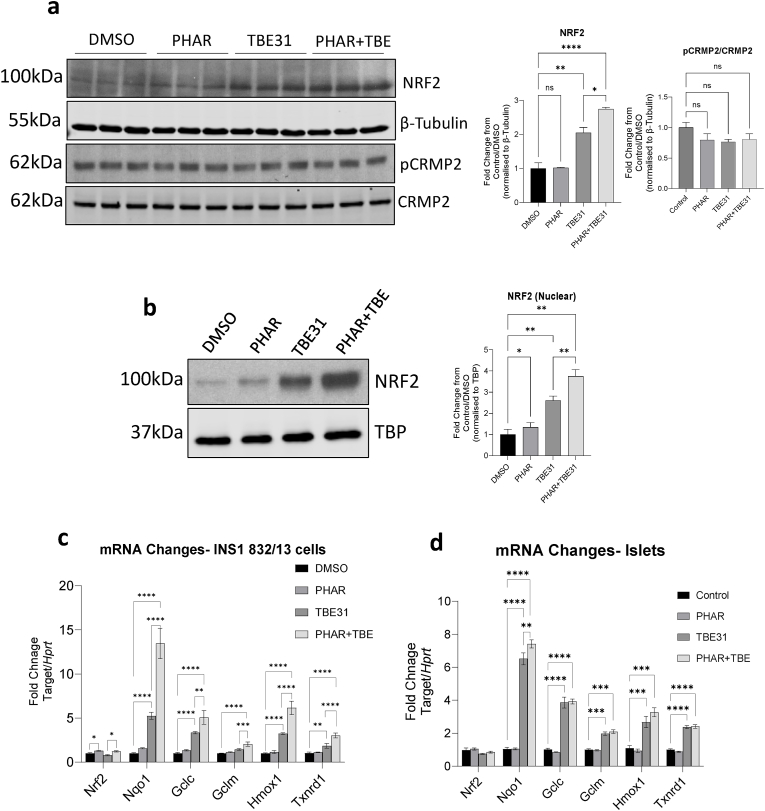
**a)** INS-1 832/13 cells were treated with DMSO, NRF2-βTrCP protein interaction inhibitor PHAR (10 μmol/l), KEAP1 inhibitor TBE31 (50 nmol/l) or PHAR + TBE31 for 24 h. Whole-cell protein lysates from three independent experiments (20 μg protein/lane) were analysed by western blotting using β-tubulin as a loading control. **b)** INS-1 832/13 cells were treated with the above agents for 24 h and cytoplasmic or nuclear fractions were extracted using NE-PER™ Kit. Nuclear fractions (10 μg protein) were separated by SDS-PAGE and NRF2 levels were analysed by western blotting using TBP as a loading control. Data from five independent experiments was analysed by paired ANOVA with Sidak's post hoc test. **c,d)** RNA was purified from INS-1 832/13 cells (n = 3), or islets from 12-week-old male C57BL/6J mice (n = 6), after 24 h treatment with the above agents and gene transcription analysed by qPCR. Data are presented as mean and standard error and analysed by one-way ANOVA with Sidak's post hoc test. *P < 0.05, **P < 0.01, ***P < 0.001, ****P < 0.0001.

Our data are consistent with previous work suggesting that GSK3 phosphorylation of the DSGIS motif in the Neh6 domain of NRF2 enhances β-TrCP binding, thereby promoting ubiquitylation and proteasomal degradation of NRF2 [13,14]. As such, we would predict that small molecules that directly interfere with NRF2-βTrCP binding should have a similar effect on NRF2 biology as inhibition of GSK3 in INS-1 832/13 cells and isolated islets. PHAR is a small molecule that specifically blocks the interaction of β-TrCP with the phosphorylated Neh6 domain of NRF2 [34]. Treatment of INS-1 832/13 cells for 24 h with 10 μmol/l PHAR alone had no significant effect on NRF2 protein levels in these cells (Fig. 4a). However, as seen with GSK3 inhibitors, NRF2 levels were higher in cells co-treated with PHAR and TBE31 compared with those treated with TBE31 alone (P < 0.05) (Fig. 4a). In addition, PHAR treatment increased the nuclear accumulation of NRF2 (Fig. 4b), both when given alone (P < 0.05) or in combination with TBE31 (P < 0.01). Finally, consistent with the GSK3 inhibition results, PHAR significantly increased the mRNA levels of NRF2-regulated genes (Fig. 4c). In INS-1 832/13 cells, PHAR augmented induction of *Nqo1* (P < 0.001), *Hmox1* (P < 0.01) and *Txnrd1* (P < 0.05) over TBE31 treatment alone (Fig. 4c). In isolated islets, PHAR enhanced induction of *Nqo1* by TBE31 (P < 0.05) (Fig. 4d) but had no significant effect on the expression of *Gclc*, *Gclm*, *Hmox1* and *Trxnd1*.

## 4 Discussion

Previous studies in neuronal and hepatic cells have shown a reciprocal relationship between GSK3 activity and NRF2 protein levels, with a simultaneous reduction in GSK3 and enhancement of NRF2-regulated gene transcription [[43], [44], [45], [46]]. That work demonstrated that GSK3 inhibitors (Lithium, TDZD-8 and SB216763) could protect neurons from oxidative insults by enhancing NRF2-directed cytoprotection. In the present paper we provide the first evidence that loss of GSK3 activity (pharmacologically or genetically) enhances nuclear accumulation of NRF2 (and the subsequent upregulation of NRF2-regulated antioxidant and detoxification genes) in the pancreatic beta cell. This link between GSK3 and NRF2 may explain the importance of each protein on beta cell mass and function [29,47,48]. It also implies that upregulation of the β-TrCP/GSK3 axis would reduce protection from NRF2 and increase the risk of progression of type 2 diabetes. Consistent with the work in other cell lines, our data imply that reducing GSK3 activity (e.g., through autocrine insulin signalling or therapeutic intervention) would extend the impact of NRF2 function by enhancing its nuclear accumulation in the beta cell, providing prolonged protection from cellular stress. Equally, reduced functionality of this pathway, for example when GSK3 activity is chronically raised due to insulin resistance, could make the beta cell more susceptible to oxidative, nitrosive or inflammatory damage, contributing to reduced beta cell mass and overt diabetes, with eventual requirement for insulin treatment.

### 4.1 Relationship between KEAP1 and GSK3

It is widely recognised that KEAP1 is a major redox sensor within the cell [49]. Our study adds to the growing evidence that the β-TrCP/GSK3 axis represents an additional KEAP1-independent mechanism to regulate NRF2 turnover. Herein, we demonstrate that GSK3 inhibition has similar effects on NRF2 in KEAP1 null INS-1 832/13 cells as in the parental INS-1 832/13 cells. Also, the increase in NRF2 activity upon treatment with PHAR (which blocks the binding of β-TrCP to NRF2 [34]) was almost identical to that caused by inhibition of GSK3, consistent with the model where GSK3 phosphorylates the DSGIS motif to enhance binding of β-TrCP and subsequent NRF2 ubiquitylation. Previous studies have also found KEAP1-independent regulation of NRF2 by GSK3, where the impact of GSK3 inhibition is significant whether KEAP1 is inhibited or not [[43], [44], [45], [46]]. That said, our data in beta cells indicate that inhibition of GSK3 in healthy cells on its own has minimal acute impact on NRF2 function, but is much more obvious when combined with a reduction in KEAP1 activity (Fig. 1, Fig. 2). This suggests that the regulation through the GSK3 phosphorylation of the phosphodegron augments, or attenuates, the primary regulation of NRF2 through the redox sensor KEAP1 in beta cells. This concords with recent work showing that a mouse with both serine residues in the DSGIS motif mutated to non-phosphorylatable alanines appeared healthy with no obvious phenotype until KEAP1 activity was suppressed, at which point NRF2 levels increased in macrophages and tissue specific hyperplasia was observed [15]. Importantly, regulation of NRF2 by GSK3 provides the opportunity for alternative environmental sensors to enhance, or prolong, NRF2-target gene expression, by reducing GSK3 activity. It remains plausible that the β-TrCP/GSK3 axis exerts tissue-specific effects, possibly assuming greater significance in tissues with relatively high metabolism such as in tumours and beta cells. Such variation is likely to be due to the different growth signals that can regulate GSK3 activity, and beta cells may be a unique case as they exhibit autocrine insulin signalling. Thus, it would be worth examining the impact of physiological and pathophysiological insulin action on the β-TrCP/GSK3 axis.

We have previously proposed that KEAP1 and β-TrCP may regulate NRF2 in different sub-cellular compartments [13]. It is widely accepted that KEAP1 is located primarily in the cytoplasm. By contrast β-TrCP may regulate NRF2 predominantly in the nucleus, in keeping with the more robust action of GSK3 inhibition on nuclear NRF2. Key components of the ubiquitylation machinery, including β-TrCP, are present within the nucleus [50,51]. Therefore, it is logical that degradation of NRF2 by β-TrCP is, at least in part, a nuclear event. This would explain the higher impact of GSK3 inhibition on NRF2 when KEAP1 is inhibited, which increases translocation of NRF2 to the nucleus. This is consistent with the previous work indicating that GSK3 inhibition can alter the ratio of NRF2 across different subcellular localisations [[43], [44], [45], [46]].

It was notable that the effect of GSK3 inhibition on NRF2 function was only seen when the majority of GSK3 activity was lost. This resembles regulation of β-catenin by GSK3, where >90% GSK3 inhibition is required to stimulate β-catenin stabilisation and accumulation, but distinct from CRMP2 where the relative amount of CRMP2 phosphorylation follows a more linear association with GSK3 activity (Fig. 1, Fig. 2, Fig. 3) rather than a threshold type effect. This implies that only a small amount of ‘normal’ cellular GSK3 activity is required to initiate NRF2 phosphorylation and turnover in healthy cells. In addition, we did not observe any evidence for selective phosphorylation of NRF2 by the GSK3α or GSK3β isoforms, which is in keeping with most GSK3 substrates that have been investigated.

### 4.2 The GSK3-NRF2 axis and gene profiles

The *Nqo1* gene is routinely measured as a functional readout for NRF2, as it is a classic ARE-containing gene promoter. We have analysed a panel of five genes which all contain at least one ARE in their promoter regions, and as such should be sensitive to NRF2 induction. In both INS-1 832/13 cells and isolated mouse islets, the KEAP1 inhibitor TBE31 induced mRNA accumulation for all five genes relative to untreated cells. Interestingly, the relative effect size on each gene (after 24 h TBE31 treatment) varied significantly (*NQO1* > *HMOX1* = *GCLC* > *GCLM* = *TXNRD1*), which was consistent between INS-1 832/13 cells and isolated islets (Fig. 1d and e), and when using KEAP1 knockout rather than TBE31 (Fig. 2c). This may reflect differences in the relative contribution made by NRF2 to the basal level of expression of each member of the ARE-gene battery that we examined.

GSK3 inhibition enhanced the mRNA levels of *Hmox1* in both INS-1 832/13 cells and islets without the need for KEAP1 inhibition (Fig. 1). This was surprising as there is very little enhancement of NRF2 nuclear accumulation by GSK3 inhibition in the absence of KEAP1 inhibition. However, this was the only one of the five ARE-driven genes we examined that responded to GSK3 inhibition and GSK3 knockdown (Fig. 2), without KEAP1 inhibition. GSK3 regulates a number of transcription factors, including c-Jun, c-Myc, CREB and NF-κB [16], and the action of GSK3 on *Hmox1* likely includes pathways independent of its regulation of NRF2, complicating the use of *Hmox1* as a readout of regulation of NRF2 by the β-TrCP/GSK3 axis.

In contrast, the regulation of *Nqo1* and *Gclm* expression by GSK3 inhibition and knockdown resembles effects on NRF2 nuclear accumulation, with enhancement only when combined with the induction by TBE31 or KEAP1 KO, and effects seen in both INS-1 832/13 cells and isolated islets (Fig. 1d and e and Fig. 2). Our data support the use of *Nqo1* and *Gclm* as the primary readouts for specific NRF2 regulation of gene transcription, and is consistent with NRF2 regulation of gene transcription in other tissues [[43], [44], [45], [46]]. There was no significant effect of GSK3 inhibition or knockdown on *Gclc* or *Tnxrd1* mRNA, perhaps due to the relatively weak induction by TBE31 or KEAP1 knockout, suggesting that the ARE sequences in these gene promoters are not as sensitive to changes in NRF2 levels, when compared with the ARE in *Nqo1*, or that they do not represent dominant regulatory elements for these genes.

### 4.3 The β-TrCP/GSK3 axis in the beta cell

Our study has not used physiological functions of the beta cell as an output. However, previous work has identified key roles for NRF2 in beta cell proliferation [52,53] and polymorphisms in *NFE2L2*, the NRF2-encoding gene, and pathway have been associated with diabetes risk [[54], [55], [56]]. Most recently, NRF2 levels were found to increase during beta cell proliferation in neonatal pancreas and to be required for proliferation, survival and beta cell mass expansion in early life, through its influence on mitochondrial ATP synthesis [53]. This implies that agents that enhance NRF2 function in beta cells could combat obesity-driven metabolic stress and protect the beta cell pool. Our data provide a potential opportunity to achieve this objective, through reduction of GSK3 activity and enhancement of nuclear NRF2. Conversely, it is conceivable that increased GSK3 activity would reduce NRF2 functional capacity. However, our data, using acute adenoviral-enforced overexpression of GSK3 in the INS-1 832/13 cell line, suggest GSK3 upregulation is not sufficient to diminish the abundance of NRF2 or its activity, at least acutely (Fig. 3), a finding that indicates further studies are required to allow a more complete understanding of the physiological relationship between GSK3 and NRF2. Type 2 diabetes is a chronic disease and results in prolonged hyperactivation of GSK3, which would be more likely to decrease NRF2 activity. In future, mouse models of type 2 diabetes treated with, or without, GSK3 inhibitors, or mice engineered to chronically express higher GSK3 in the pancreatic beta cells, could confirm the impact of long term raised GSK3 activity. Besides difficulty in modelling the effects of chronic disease on GSK3 activity, it is equally plausible that since NRF2 requires prior phosphorylation by an alternative protein kinase to permit recognition by GSK3 [16,57,58], the priming processes may limit the ability of GSK3 to phosphorylate NRF2 and so create the phosphodegron. There are four potential ‘priming’ serine residues C-terminal to the DSGIS motif in NRF2, and one or more of these may be crucial for phosphorylation of the DSIGS motif by GSK3. The priming requirement of NRF2 deserves study since activation of this mechanism would antagonise NRF2 function in the beta cell, while the inhibition of the priming pathway would be an alternative therapeutic option to GSK3 inhibition to enhance NRF2 protection in the beta cell. It may be a preferable option since there is concern that chronic inhibition of GSK3 would have harmful side-effects due to its key roles in many fundamental cellular processes.

## 5 Conclusions

In summary, we propose that GSK3 regulation of NRF2 enhances or prolongs the NRF2 response by enhancing accumulation in the nucleus of beta cells, most noticeably augmenting the main regulation by the redox sensor, KEAP1. This degradation pathway likely targets NRF2 in the nucleus, and hence impacts the regulation of key stress response genes, in particular *Nqo1* and *Gclm*. In type 2 diabetes, a chronic progressive disease, defects in this pathway would impair beta cell defences against nutrient and inflammatory stress, worsening decline in the functional beta cell mass and accelerating progression toward the need for insulin treatment.

## Funding

This work was supported by a 10.13039/501100000361Diabetes UK research grant [grant ref:20/0006178] awarded to CS, JC, JDH and AMN; MICINN grants PDC2021-121421-I00, PDC2022-133765-I00 and PID2022-141786OB-I00 awarded to AC. The funder had no input in study design; in the collection, analysis and interpretation of data; in the writing of the report; and in the decision to submit the article for publication.

## Declaration of interest statement associated with the following submission

AC is founder of the Phamaceutical company Servatrix Biomed S.L. We can confirm that all other authors in the list above have confirmed that they do not have any financial/personal interest or belief that could affect their objectivity in this submission.

## CRediT authorship contribution statement

**Chinmai Patibandla:** Writing – original draft, Methodology, Investigation, Formal analysis, Data curation. **Lidy van Aalten:** Investigation. **Albena T. Dinkova-Kostova:** Writing – review & editing, Conceptualization. **Tadashi Honda:** Writing – review & editing, Resources. **Antonio Cuadrado:** Writing – review & editing, Resources, Conceptualization. **Raquel Fernández-Ginés:** Writing – review & editing, Resources. **Alison D. McNeilly:** Writing – review & editing, Project administration, Investigation, Funding acquisition. **John D. Hayes:** Writing – review & editing, Funding acquisition, Conceptualization. **James Cantley:** Writing – review & editing, Project administration, Funding acquisition, Conceptualization. **Calum Sutherland:** Writing – review & editing, Supervision, Project administration, Funding acquisition, Conceptualization.

## Data Availability

Data will be made available on request.
