# Supplementary material for: Inhibition of glycogen synthase kinase-3 enhances NRF2 protein stability, nuclear localisation and target gene transcription in pancreatic beta cells
Source: Redox Biol. 2024 Mar 7;71:103117. doi: 10.1016/j.redox.2024.103117 (PMC10950707; doi:10.1016/j.redox.2024.103117)
Supplement: Multimedia component 1 [file mmc1.pdf]

Supplementary Figure 1: Time-dependent adenoviral GSK3 overexpression in INS-1 832/13 cells

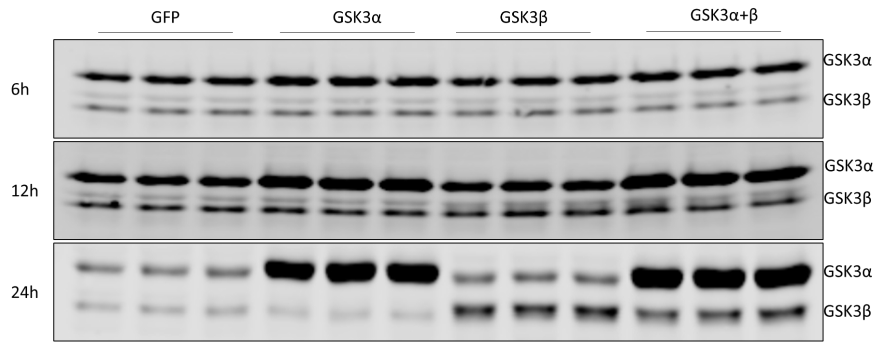

Supplementary Figure 1: INS-1 832/13 cells were transduced with adenoviral particles expressing GSK3α or GSK3β under the CMV promoter. Expression levels of total GSK3 at 6, 12 or 24 hr post-transduction were analysed by western blotting.

Supplementary Figure2: NE-PER^{TM} Cytoplasmic and Nuclear extraction kit_Purity of fractions

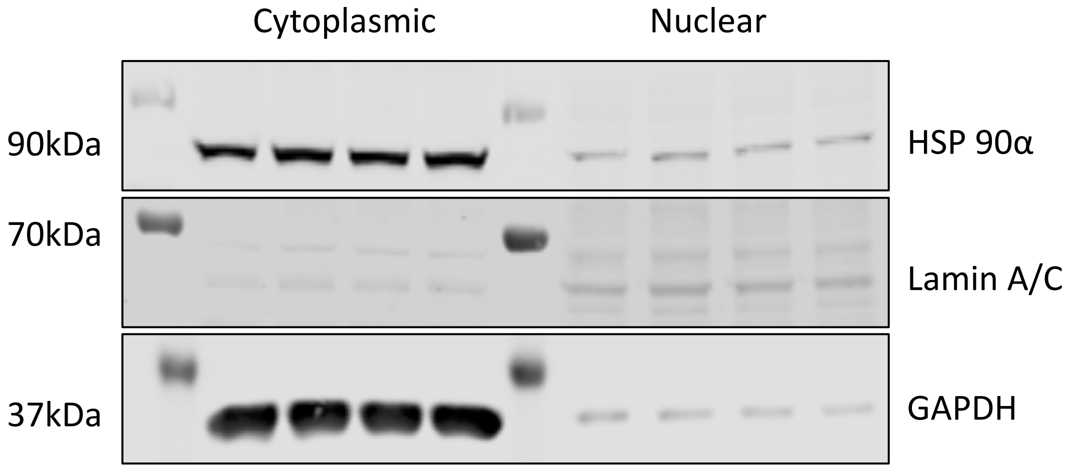

Supplementary Figure 2: INS-1 832/13 cells were treated for 24 h with DMSO, CT99021 (5 µmol/l), TBE31 (50 nmol/l) or CT99021 +TBE31, and then cytoplasmic and nuclear protein extracts prepared. Samples of either cytoplasmic fractions (20 µg protein/lane) or nuclear fractions (10 µg protein/lane) were separated by SDS-PAGE. The quality of fraction preparations was checked by cytoplasmic (HSP90α, GAPDH) or nuclear (Lamin A/C) markers

Supplementary Figure3: Gsk3 inhibitor CT99021 treatment for 36h

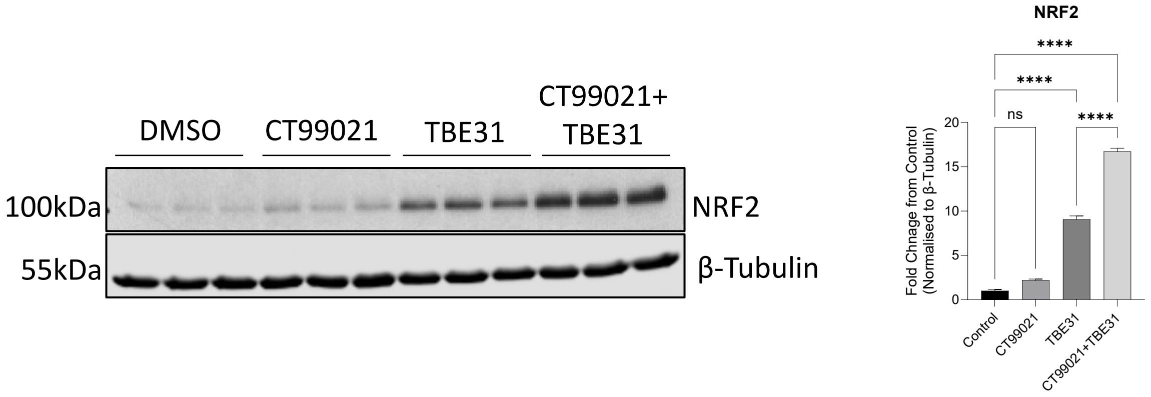

Supplementary Figure 3: INS-1 832/13 cells were treated for 36 h with DMSO, the GSK3 inhibitors CT99021 (5 µmol/l), the KEAP1 inhibitors TBE31 (50 nmol/l) or combinations CT99021+TBE31. Whole-cell protein lysates from three independent experiments (20 µg protein/lane) were analysed by western blotting using β-Tubulin as a loading control. Data were analysed by paired ANOVA. Data are presented as mean and standard error and analysed by one-way ANOVA with Sidak's post hoc test. ****P<0.0001.
